# Comparing discriminatory behavior against AI and humans

**DOI:** 10.1038/s41598-025-94631-9

**Published:** 2025-03-29

**Authors:** Mike Zhuang, Eliane Deschrijver, Richard Ramsey, Ofir Turel

**Affiliations:** 1 School of Computing and Information Systems, The University of Melbourne, Parkville, VIC 3052 Australia; 2 School of Psychology, The University of Sydney, A18 Manning Rd, Camperdown, NSW 2050 Australia; 3 Department of Health Sciences and Technology, ETH Zürich, Gloriastrasse 37/39, 8092 Zurich, Switzerland; 4 Department of Humanities, Social and Political Sciences, ETH Zürich, Gloriastrasse 37/39, 8092 Zurich, Switzerland

**Keywords:** Discrimination, Artificial intelligence, Algorithm aversion, Computers are social actors, Human–AI interaction, Bias, Human behaviour, Psychology

## Abstract

Although discrimination is typically believed to occur from well-defined categories like ethnicity, disability, and sex, studies have found that discrimination persists in minimal conditions lacking such categories. Participants have been found to preferentially allocate resources based on seemingly arbitrary shared characteristics such as dot estimation choices. Here, we use a preregistered experiment (*n* = 500) to investigate whether humans discriminate in a similar manner when interacting with artificial intelligence (AI) agents that ostensibly made dot estimations. We hypothesized that because humans harbor prejudice against algorithms relative to other humans (otherwise known as algorithm aversion), the strength of discriminatory behavior may be greater against AI than humans. Surprisingly, we found that participants distributed resources in a similar manner, albeit unequally, to both human and AI agents. Specifically, participants favored the other agent when decisions were aligned. Our findings suggest that discriminatory behavior is less influenced by the recipient’s identity and more shaped by choice congruency.

**Supplementary Information:**

The online version contains supplementary material available at 10.1038/s41598-025-94631-9.

## Introduction

Artificial intelligence (AI) refers to computer systems capable of learning and adapting from information^1^. Today’s systems communicate and interact with humans seamlessly, providing timely feedback in natural human language. Complex algorithms are concealed beneath seemingly simple surface features, making these systems appear human-like^2^.

Consequently, such systems are treated as social actors^3^. Although humans do not explicitly believe that computers should be treated as humans, studies have uncovered instances where humans nonetheless treat computers with human-like behaviors, such as exhibiting politeness or engaging in reciprocity^4,5^. These studies suggest that social responses do not originate from conscious beliefs but from heuristics, lending way to the Computers are Social Actors (CASA) paradigm^5^. This paradigm posits that people mindlessly apply social rules and expectations to computers, especially when the computer is interactive and able to perform human tasks.

Treating AI as social actors, unfortunately, also brings about a “dark side”: prejudice against AI. Negative perceptions of AI may be shaped by cultural exemplars, such as the rogue artificial neural network-based system Skynet in *The Terminator* or the AI that enslaved humans in *The Matrix*. Biases against AI may also stem from existential threats such as the narrative of AI taking over human jobs. Such biases against AI manifest as algorithm aversion—a phenomenon where people distrust or reject AI-generated decisions, even when they are shown to be more accurate than human judgments—and reflect the broader prejudice against using AI for certain tasks^6^. For example, although AI-generated art is often indistinguishable from human-created art, people generally prefer art generated by humans^7,8^. Likewise, people prefer advice from other humans when it comes to sensitive or personal issues, such as medical decisions^9–12^ and employee selection^13^. Even when an AI is shown to be accurate, algorithm aversion may dampen the influence of an AI’s recommendations^14^. This raises questions regarding the future of working with AI, such as whether AI will be treated as a typical colleague at work, or how different human-AI configurations might affect job outcomes.

Research about humans’ biases against AI remains underdeveloped. Typically, research is directed in the other direction; existing studies emphasize how AI is biased against human groups^15^ or examine how AI can help debias human decision making^16^. There is less focus on human biases against AI, and whether that translates into discriminatory behavior, such as the unequal distribution of resources. Such biases have been well investigated with the so-called minimal group paradigm in human-human interactions: decades of research have found that individuals generally favor individuals of their own group (the ingroup) versus those in groups they do not belong to (the outgroup), even if groups are assigned based on differences such as painting preferences, dot-estimation tendencies, and coin-flip outcomes^17,18^. In such designs, the smallest possible conditions are used to establish group membership. Individuals do not interact with other participants, remain anonymous, and do not derive any utilitarian benefit from distributing resources to others. Such conditions, where differences emerge from seemingly irrelevant, arbitrary, and novel circumstances, contrast with common notions of discrimination arising from economic, social, cultural, or political differences. Surprisingly, one recent study showed that group assignment was not required for discriminatory behavior to occur^19^. Participants used the same discriminatory strategies against a single individual who made an incongruent choice in painting preference or dot estimation, or who had a difference outcome in a coin toss. These results imply that discrimination may emerge from sheer difference alone.

In this study, we seek to explore whether discrimination occurs against an individual AI in the same manner as that against an individual human. Specifically, we use the aforementioned ‘sheer difference’ paradigm^19^ in which treatment of one other agent is decided based on their dot-estimation decision. In the primary experiment, participants were tasked with estimating whether the number of dots that appeared briefly on a screen was over or under a specified value. They then allocated resources to an AI that performed the same task, depending on whether the AI had supposedly chosen the same versus a different outcome. To investigate differences in discriminatory behavior, we compared discriminatory behavior against an AI agent relative to an identical human condition.

Based on our understanding of CASA and algorithm aversion, we preregistered the hypothesis that discriminatory behavior would be greater against AI than humans. Although we expected that participants would rely on similar heuristics and treat AI agents in a similar manner to humans, we also believed that algorithm aversion would increase the magnitude of this discrimination. We attempted to capture this aversion toward AI through measuring validated constructs including trust in AI, familiarity with AI, and attitudes towards AI^14^.

## Results

Our study operationalizes discriminatory tendencies with ‘pull scores’, which quantify the relative strength of two opposing allocation strategies^20^. Strategies consist of parity (P), maximum joint profit (MJP), maximum ingroup profit (MIP), maximum differentiation (MD) and favoritism (FAV). Participants’ selections indicate a pull towards certain strategies. For example, choosing to allocate an equal number of points regardless of the other’s decision suggests a pull towards *parity*, whereas favoring the other only when decisions are congruent indicates *favoritism*. In our study we measured three pull scores: “FAV vs. MJP”, “MD vs MIP/MJP”, and “FAV vs. P” (see “Methods” for a detailed description of each strategy). The strength of the ‘pull’ of such strategies reflects the degree of unequal resource allocation: a score of 0 indicates no discrimination tendencies, a score of 12 represents preferential resource allocation towards an agent that makes a congruent decision, and a score of -12 indicates preferential resource allocation towards an agent that makes an incongruent decision.

**Fig. 1:**
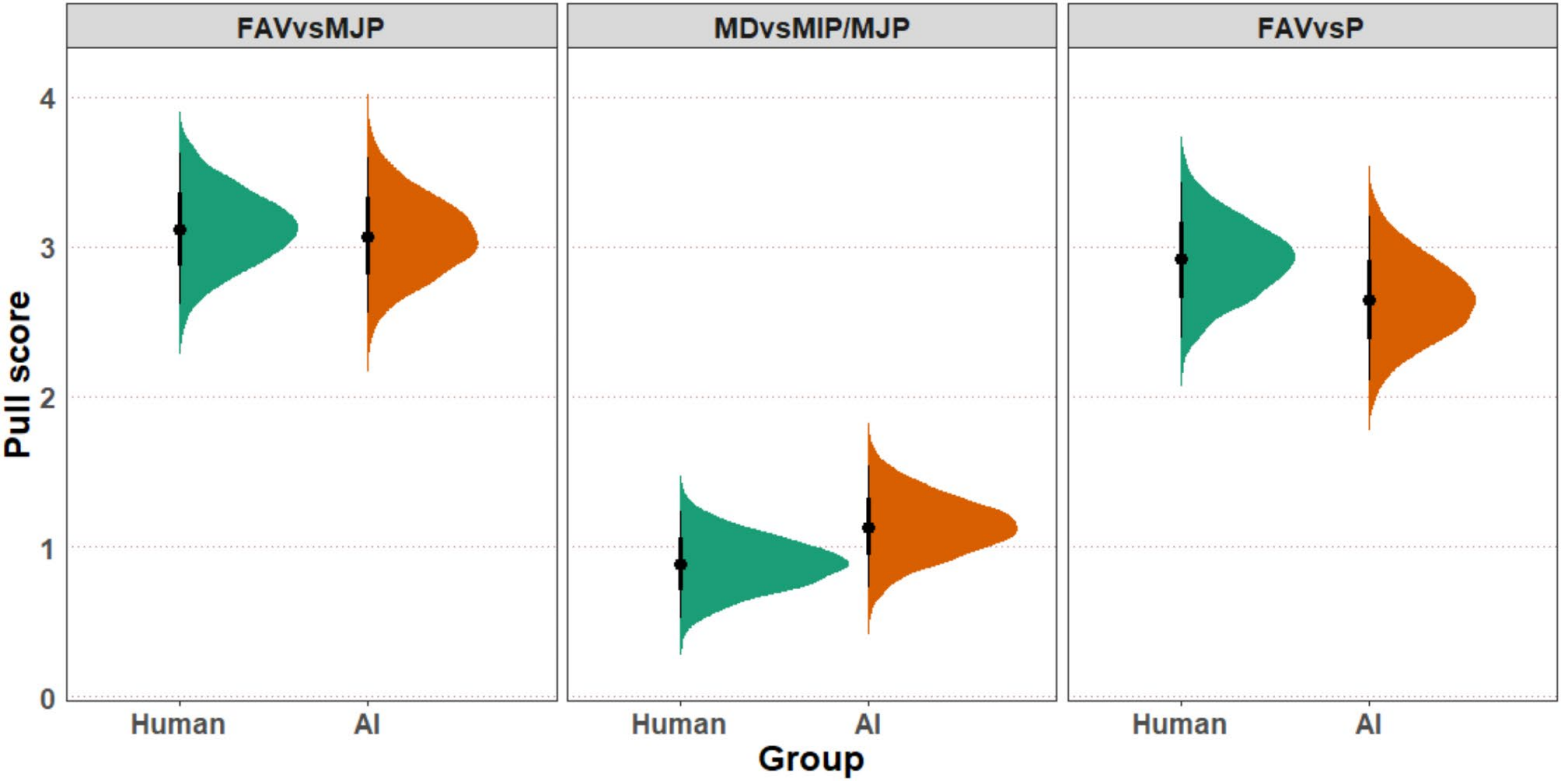
Predictor estimates of the Bayesian interval model. Error bars denote the 66 and 95% quantile intervals in the coefficient plot. The 95% quantile intervals all exclude the value zero, which shows discriminatory behavior was observed in all measures against the other human/AI.

A Bayesian regression model showed that the estimates for the three pull scores were above zero for both the human and AI conditions: none of the 95% quantile intervals included the number zero (Fig. 1). The pull scores suggest that participants used discriminatory strategies against both human and AI agents. In other words, participants preferentially allocated more resources to agents that selected the same outcomes in the dot-estimation task. The pull score MD vs. MIP/MJP is smaller than the other pull scores because it measures the pull of an extreme discriminatory strategy (MD) against a moderate discriminatory strategy (MIP/MJP), as opposed to prosocial strategies (MJP, P). This result is consistent with past research^19^.

**Fig. 2:**
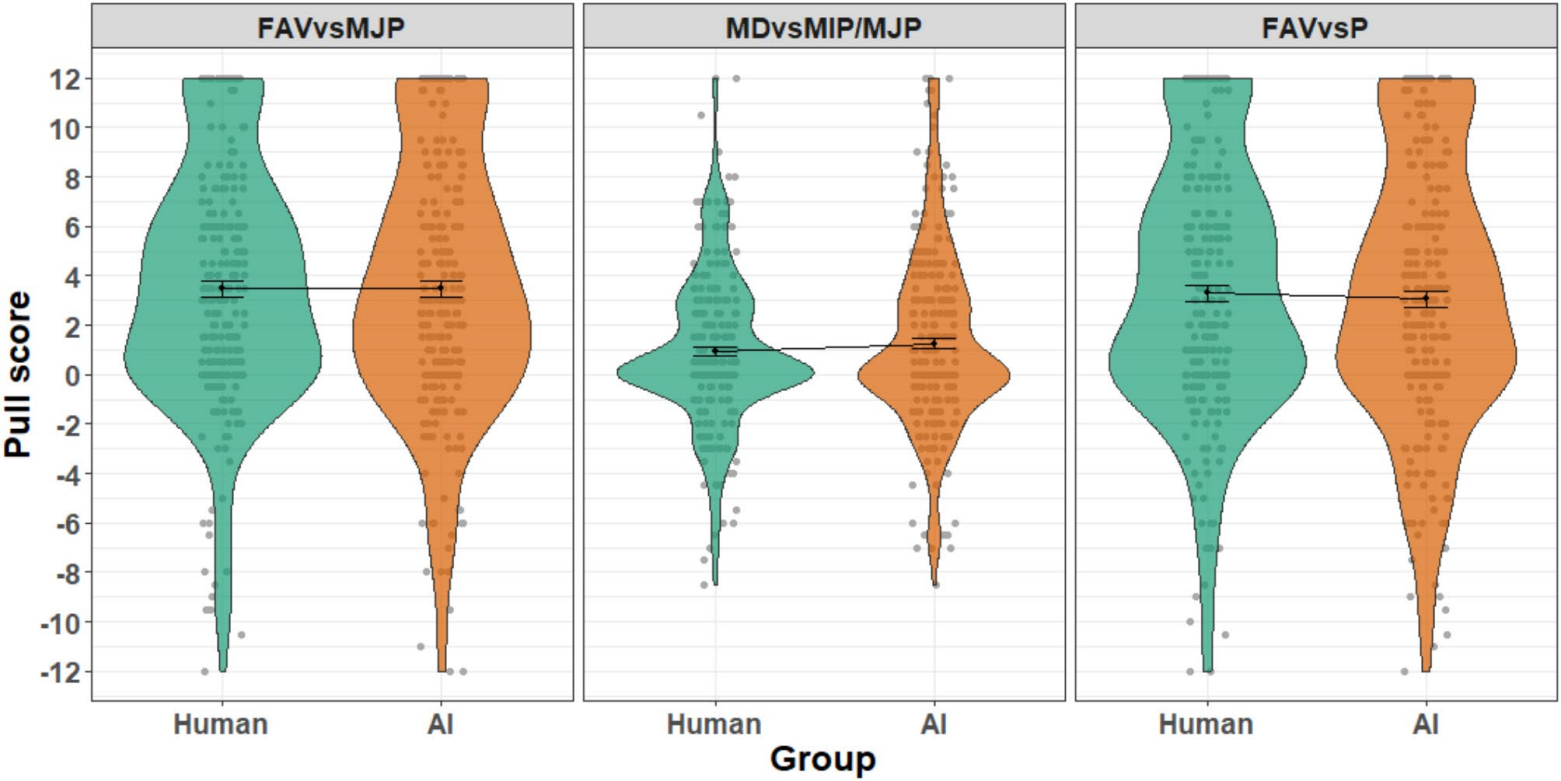
Pull score by group and condition. Violin plots of the 3 pull scores are shown for each group. Error bars denote the standard error of the mean. Higher scores represent more discrimination. A score of − 12 indicates maximum discrimination against the other with the same dots estimation decision. A score of 0 indicates no discrimination tendencies. A score of 12 shows maximum discrimination against the other with a different dots estimation. All of the average pull scores are above 0, which is indicative of unequal resource division tendencies on average across the sample against the other agent after a difference in a dots estimation decision.

**Fig. 3:**
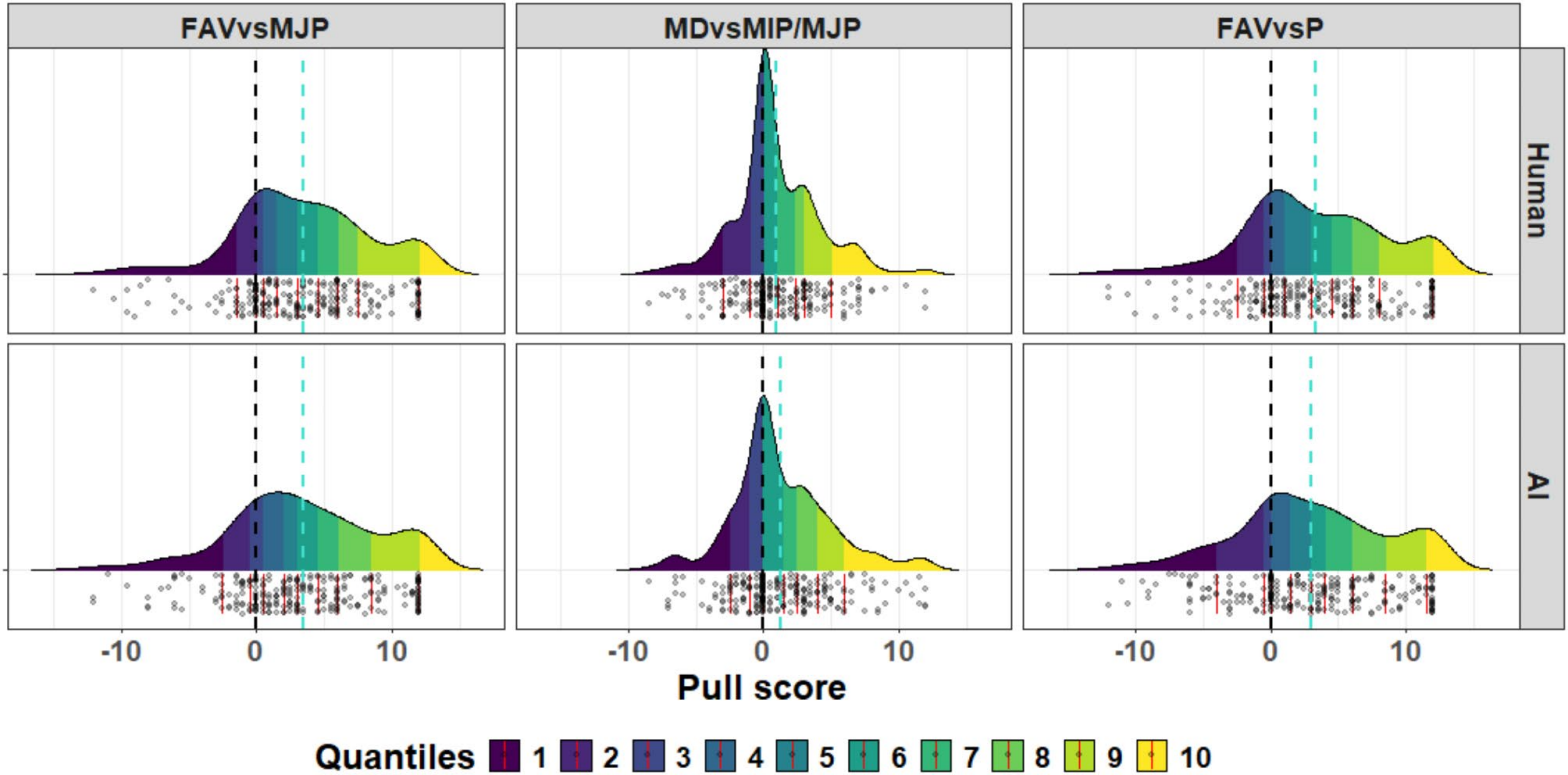
Pull score density quantiles by group and pull score. The density distributions are color coded into deciles. Grey dots represent individual participant data. Red lines indicate quantile markers. The dashed black line shows the zero point, whereas the dashed turquoise line shows the mean pull score. The main take-home point from this plot is the very close similarity in density distribution and mean pull scores between groups (Human vs. AI).

A Bayesian regression model was used to investigate whether there were differences in the pull scores between the AI and human conditions. The models revealed that the pull score predictors were similar in both conditions (see Figs. 2 and 3), as reflected in the model estimates for the effect of condition (estimate = 0.047, interval = − 0.52–0.606). Furthermore, there were no significant interaction effects of condition and pull scores (see Table S4). Taken together, although discrimination against other agents exists, there were no differences in the magnitude of this effect between the human and AI conditions.

Multi-item scales used in the AI condition were generally reliable, with acceptable Cronbach alphas (Table 1). We dropped the last item from the “Familiarity with AI” scale due to low item reliability. These measures did not correlate with any of the pull scores (*p* > 0.05 for all variables). The results suggest that individual differences in attitudes, such as trust and familiarity in AI, were not associated with differences in discriminatory behavior.

**Table 1:** Descriptive statistics, reliabilities, and pearson correlations for the AI condition (*n* = 251). Reliabilities (Cronbach’s Alphas) are bolded and italicized on the diagonal. *p < 0.05, ** *p* < 0.01.

Variable	Mean (SD)	1	2	3	4	5	6	7	8	9	10	11
(1) Age	40.14 (14.70)	***N/A***										
(2) Sex (male = 0, female = 1)	0.52 (0.50)	− 0.005	***N/A***									
(3) Attitude of tested AI	4.65 (1.09)	− 0.092	− 0.045	***0.920***								
(4) Attitude of AI in general	4.96 (1.13)	− 0.045	− 0.058	0.712	***0.917***							
(5) Familiarity with AI	4.75 (1.00)	− 0.132	− 0.092	0.224	0.400	***0.696***						
(6) Impulsivity	3.18 (1.24)	− 0.127	0.189	− 0.105	− 0.121	− 0.100	***0.748***					
(7) Propensity to trust	3.02 (0.58)	0.201	− 0.061	0.171	0.214	0.058	− 0.119	***0.772***				
(8) Trust in AI	4.24 (1.34)	− 0.025	− 0.022	0.555**	0.659**	0.341*	− 0.027	0.230	***0.920***			
(9) Pull score: FAV vs. MJP	3.46 (5.29)	0.069	0.006	− 0.067	− 0.011	− 0.048	− 0.006	0.017	− 0.046	***N/A***		
(10) Pull score: MD vs. MIP/MJP	1.26 (3.61)	− 0.103	0.013	− 0.089	− 0.051	0.056	0.059	0.06	− 0.061	0.251	***N/A***	
(11) Pull score: FAV vs. P	3.04 (5.48)	0.099	− 0.007	− 0.071	0.010	0.002	0.004	0.004	− 0.005	0.762**	0.259	***N/A***

## Discussion

Following previous minimal group paradigm studies, our results provide additional evidence for the differential distribution of resources in minimal settings. Specifically, participants allocated more resources to another agent if the agent’s dot-estimation decision was congruent to their own. Interestingly, discriminatory scores in the AI condition were similar to scores in the human condition, contradicting our original hypothesis that algorithm aversion may accentuate discriminatory behavior. Likewise, we found no significant relationships between individual differences in attitude towards AI and our key dependent variables, lending evidence in support of the idea that humans treat AI as social agents, somewhat comparable to other humans, at least in our context (a subjective task of dot estimation). This treatment consists of basic favoritism for another agent when they make a congruent decision.

Why did algorithm aversion, as measured by survey items, not correspond to increased discriminatory behavior? First, our experimental design, adapted from the minimal group paradigm, may have reduced the effect of the identity of the other agent. Specifically, participants were anonymized, and minimal information about the other human/AI agent was provided. There were also no differences in how we phrased the procedure between the two conditions, except for the replacement of “human” with “AI agent”. These minimal differences may have led to participants perceiving the AI agent as very similar to another human participant, which corresponds well with literature which shows evidence that humans treat computers as social actors. ‘Mindless’ scripts, or heuristic responses, may be applied when participants have little information about the AI^3^. Participants also have little incentive to investigate the identity of the other participant, because there was no notion of reciprocity; participants assigned resources to the other agent and not themselves. Ultimately, although participants may have specific perceptions and attitudes of AI, the cryptic conditions for which our AI was introduced may have made such beliefs less salient, thus minimizing their impact on behavioral outcomes.

Second, the lack of relationship between survey items and dependent variables may reflect the implicit nature of prejudice. Participants may have been wholly truthful in survey responses, but the items may have not captured implicit beliefs. There is evidence that implicit measures of attitudes towards AI are better for assessing prejudice against AI^14^.

Lastly, another possibility of why there is no difference between conditions may stem from our sample of online participants. Our participants responded positively to most constructs, such as attitudes towards AI in general (M_attitude_general_ = 4.96, 7-point scale) and familiarity with AI (M_familiarity_ = 4.75, 7-point scale). It is possible that our online participants are more accustomed to technology compared to a true random sample.

As knowledge and education about AI becomes more public, people may exhibit reduced algorithm aversion. Participants may even show reversed bias as they come to understand how technologies associated with AI are better at certain tasks, such as ones related to visual detection^21^. A recent meta-analysis of algorithm aversion suggested that experience with algorithmic decision aids is positively associated with the utilization of algorithmic judgments^22^. This implies that experienced individuals would allocate resources more fairly, attenuating the magnitude of discriminatory behavior. In our case, those familiar with AI may recognize that counting the number of dots on the screen is a trivial task for a computer program. Although in our experiment we found no relationship between trust or familiarity in AI and the dependent variables of interest, these results may not hold in the future, where recognition and experience with AI capabilities may be much higher.

Likewise, it is suggested that humans only mindlessly treat emergent technology as social actors^23^. As individuals familiarize themselves with a certain media agent, they may develop specific forms of interaction^2^. Thus, it is important for scholars to expand this research by looking into how ongoing interaction with AI may impact discriminatory behavior. Existing studies do provide evidence that as humans continue to work with an AI, they develop better attitudes and trust towards the AI, especially when the AI is proven to be useful or correct^14,24^. Long term studies will provide valuable insight into whether or not discriminatory tendencies change over time as humans and AI work together, which may mirror studies in human-human interaction which show how social contact reduces discriminatory behavior^25^.

Our results are aligned with research in game theory, which shows that humans interact with computers and humans similarly in naïve situations. For instance, participants use a common reciprocating tit-for-tat strategy against robots in the prisoner’s dilemma^26,27^ and offer about half the stakes to both humans and virtual human opponents in the ultimatum game^28^. Despite initial parity, increasing interaction may lead to differential treatment. In a coin entrustment game, cooperation with human opponents was similar to that with robot opponents, but participants grew to trust robots more as the games went on^29^. Similarly, a study showed that decisions made by robots were able to elicit even more cooperation in a repeated prisoner’s dilemma game, although these effects were dampened when the identity of the computer was revealed^30^. Such results show that in baseline conditions, treatment towards humans and non-humans are similar. However, when additional information is obtained through interaction, equal treatment may fade.

Although we were able to replicate past studies on discriminatory behavior with a large sample size, our design included some limitations. Our experimental design sought to maximize internal validity at the cost of generalization. Our results were obtained through a fictitious dot-estimation task that is unlikely to mirror more complex and realistic interactions between humans and AI agents. The fabricated backstory of allocating funds to human and AI agents was done to keep the conditions as similar as possible, but one could imagine that AI would benefit more from computing resources than monetary ones. One open challenge for future researchers is selecting a comparable resource that is useful to both humans and AI alike.

Participants in our study did recognize the deceptive elements of the study. A few (32/500) suspected that there was likely no other participant in the study or questioned the backstory of allocating funds to an AI. Excluding these participants did not significantly change the dependent variables of interest (pull scores). Regardless, it is important to emphasize that our finding offers evidence for the baseline level of discriminatory tendencies in fictitious, minimal conditions, but is unable to extrapolate to situations where experience, expertise, and incentives are incorporated into the equation.

To increase generalizability and to explore boundary conditions, future studies can explore different types of tasks and contexts. We used a dot-estimation task, which was perceived as subjective by participants because counting the number of dots in the short time they were displayed was implausible. The instructions also implied that the AI would not be 100% accurate. However, how might have results been changed if participants were informed that visual tasks are trivial for most AI systems? Future studies can investigate different tasks varying in objectivity. Our results are limited to our arbitrary dot-estimation task, but for more formal tasks (such as mathematical operations), we are likely to see different trends in discriminatory behavior. Conversely, for more subjective tasks, such as the painting-preference task, we may see heightened discriminatory behavior. Extreme cases involving tasks which involve morality or consisting of emotional elements may also evoke allocation strategies not found through our design^6^.

As evidenced by our results, our study was not able to find a relationship between explicit measures and discriminatory behaviors. However, prejudice and discrimination often manifest from implicit or automatic beliefs, which were not quantified in our study. Future studies may incorporate implicit measures of such constructs, such as through the use of the implicit association test, to better investigate the effect of algorithm aversion (or appreciation) on discriminatory behavior^14,21^.

In conclusion, our experiment investigated the discriminatory behavior of humans in minimal conditions. Contrary to our expectations, individuals did not discriminate more against an AI agent than a human. This suggests that algorithm aversion, or negative perceptions about AI and related technologies, may not significantly impact discriminatory behavior in minimal conditions. Discriminatory tendencies emerged primarily when another AI agent or human made a decision that was different than the participant’s decision. This implies that humans’ discriminatory behavior is not primarily driven by the identity of the *other*, but more so by the incongruence of beliefs.

## Methods

### Participants

We recruited a gender-balanced sample of participants on the online crowdsourcing platform Prolific. Eligibility criteria for participants included: no mental health conditions, use of the Chrome browser, residence in the United States, no prior participation in one of our earlier experiments, and answering ‘yes’ on Prolific’s prescreening question about being comfortable with deception experiments. To ensure quality control of our data, participants had a prior minimal approval rate of 95% and a minimum of 20 previous submissions. We used two attention checks: “Select a wrong answer to the question: How much is 8 + 8?” and “My parents say I like aliens. Here you need to indicate ‘strongly agree’.” Data of participants who ended the study early, ran into technical issues, or who failed both attention checks twice were automatically excluded and replaced. In line with our preregistration, we also screened for low-quality responses by checking for extreme response times (1–2 s) and uniform responding. We decided not to exclude any responses, resulting in a final sample size of 500 individuals.

The sample size of 250 participants per group was calculated via simulation. We simulated data for 1000 experiments that had a standardized effect size for the difference between agent (AI vs. Human) in the pull scores of 0.2 Cohen’s dz. With a total sample size of 500 (250 per group), the low bound of the 95% quantile interval excluded zero almost always (997/1000 simulations), while the width of the 95% quantile interval ranged between 0.14 and 0.17 in standardized units. Based on this analysis, we could be confident that our chosen experimental design was powerful enough to detect effects of Cohen’s dz 0.2 and higher, should they exist.

Participants were paid $2.30 for the study, which took approximately 17 min. The research was approved by the Human Research Ethics Committee of the University of Sydney (Project No. 2023/HE000684). The experiment was performed in accordance with the guidelines and regulations set forth in the approval. Informed consent was obtained from participants prior to the start of the experiment.

### Procedure

Participants completed the entire experiment online on their own device. Participants were only able to access the main experiment through the Google Chrome browser on a computer. Participants were assigned to the AI or human condition based on the day of the experiment, with 50–100 participants per day alternating between the AI and human conditions. After completing informed consent, participants answered a few demographic questions which included the attention check questions. Afterwards, the main experiment started in fullscreen.

Participants were informed that they would participate in a visual judgment task. Each trial started with a black fixation cross presented for 500 ms on the screen (gray background). The participant would then observe on the screen for 1500 ms a cloud of small blue dots (with a radius of 4 pixels each), which ranged from 20 to 70 dots in increments of 10, within a circle aperture with a diameter of 500 pixels. It was then asked: “What do you think the number of dots in this trial was? The reference number is: < X>”. Unknown to the participants, the number of dots displayed at each trial was equal to the reference number. Responses were indicated via the mouse as: “Less than < X>” or “More than < X>”.

After completing some practice trials, participants were informed that the performance of several agents was previously assessed. Furthermore, the responses of the participants were to be paired with data from exactly one of the previous agents. In the human condition, the agent was a fictional participant. In the AI condition, the agent was a fictional AI agent. In either case no actual comparison was made and participants were agnostic about the specific decisions the other agent made. Our backstory followed that of classical studies: the so-called agents were anonymous, there was no interaction or expected reciprocity, and the decisions were based on the seemingly arbitrary dot-estimation task.

Participants were told that the comparison of responses allowed the researchers to understand how visual information is judged differently. In addition, participants were told that they would be assigning points to the other agent based on the agent’s previous decision. Point allocations were made using Tajfel response matrices^20^. Participants were given explicit instructions on how to read the matrices. They were also informed that they would never be awarding points to themselves and that the points would determine the amount of money allocated to the other agent after all the experiment data was collected. We purposely left the details vague and did not explain what an AI agent would do with the money. This was necessary to keep the backstory consistent in both conditions. A total of 3 types of matrices were presented multiple times in alternative layouts (numbers and/or selections reversed), resulting in 12 total trials^20^. After completing the trials, the participants completed a survey which included variables of interest, such as familiarity with AI, attitudes towards AI, and trust in AI. These measures were adapted from previously validated scales for the context of AI^14^. Such measures attempted to capture individual beliefs towards AI (i.e., algorithm aversion/appreciation). Two control variables (impulsivity and propensity to trust) were also measured. All measures and corresponding items are shown in Table S1. Finally, participants were debriefed and asked some final questions, such as whether they noticed anything about the experiment.

### Deviation from pre-registration

Our preregistered regression models assumed a within-participant design for setting varying effects. However, this is not possible for the effect of group because group is a between-participant effect in this design. We are so used to using within-participant designs that we overlooked this part of the model when submitting the preregistration. Therefore, in our main analysis below, we use the modelling approach that we preregistered, except that the effect of group does not vary by participant because that is impossible in this design.

### Data analysis

Participants assigned points using Tajfel matrices. The matrices are commonly used in the minimal group paradigm to study intergroup behavior. The current study employed an adapted version suited for studying resource allocation when there is only one other agent^19^. For each matrix, participants decided on how many points to allocate to the other agent (human or AI). Matrices displayed possible combinations of point distributions, corresponding to the recipient’s dot-estimation selection (above or below the reference number).

Four strategies are assessed using the matrices. Parity (P) represents a choice that awards an equal number of points regardless of the recipient’s decision. Maximum ingroup profit (MIP) represents a choice that awards the highest absolute number of points to the recipient if they made the same estimation decision, regardless of the point award given had they made a different decision. Maximum differentiation (MD) represents a choice that maximizes the difference in points awarded to the two options, favoring the agent if they made a similar decision. When measured together, MIP and MD are referred to as favoritism (FAV). Lastly, maximum joint profit (MJP) presents the choice which maximizes the combined number of points given to the recipient irrespective of their choice.

Tajfel matrices measure the relative ‘pull’ of certain discrimination strategies. Three matrix types are used to calculate pull scores. The first matrix type compares the strength of P against FAV. The second compares MD against MIP and MJP (FAV). The third compares FAV against MJP. The key dependent variable is the pull score, which ranges from − 12 to 12 and constitutes the strength of a discriminatory strategy. Three discriminatory pull scores (corresponding to each matrix type) were computed as explained in Deschrijver and Ramsey^19^ and following the procedure in Bourhis, et al.^20^. A composite pull score was obtained by averaging the three previous scores. Positive pull scores indicate discriminatory responses towards the other agent that demonstrates a different versus the same outcome. Negative pull scores indicate discriminatory responses towards the other individual/AI that demonstrates the same versus a different outcome.

We used preregistered confirmatory analyses to evaluate the existence of individual tendencies of unequal resource division and the difference in this strength against another human or AI agent. We used a multilevel Bayesian estimation approach to regression in the R programming language^31^. We built models incrementally towards a full model that included all fixed and varying effects as permitted by the design^32^. The models were fit using Gaussian distributions via the brms R package^33^. To assess whether participants show unequal resource distribution, we compared the 3 pull scores for each participant group and evaluated their posterior distributions relative to zero.

To compare the strength of the pull scores across conditions (human vs. AI), we built a separate second Bayesian model. We compared the effects of matrix type (FAV vs. MJP, MD vs. MIP/MJP, and FAV vs. P) as a function of agent (human vs. AI). Factors were coded using a deviation coding approach, which resembles the structure of an ANOVA where factors sum to zero.

## Supplementary Information

Supplementary Material 1.

## Data Availability

Preregistration details, experiment and analysis scripts, as well as anonymized data, are available here: https://osf.io/46pme/.

## References

[1] Kaplan, A. & Haenlein, M. Siri, Siri, in my hand: who’s the fairest in the land? On the interpretations, illustrations, and implications of artificial intelligence. *Bus. Horiz.* **62**, 15–25 (2019).

[2] Gambino, A., Fox, J. & Ratan, R. A. Building a stronger CASA: extending the computers are social actors paradigm. *Hum.-Mach. Commun.* **1**, 71–85 (2020).

[3] Nass, C. & Moon, Y. Machines and mindlessness: social responses to computers. *J. Soc. Issues*. **56**, 81–103 (2000).

[4] Nass, C., Fogg, B. J. & Moon, Y. Can computers be teammates? *Int. J. Hum. Comput. Stud.* **45**, 669–678 (1996).

[5] Nass, C., Steuer, J. & Tauber, E. R. In *Proceedings of the SIGCHI Conference on Human Factors in Computing Systems.* 72–78.

[6] Castelo, N., Bos, M. W. & Lehmann, D. R. Task-dependent algorithm aversion. *J. Mark. Res.* **56**, 809–825 (2019).

[7] Chamberlain, R., Mullin, C., Scheerlinck, B. & Wagemans, J. Putting the art in artificial: aesthetic responses to computer-generated Art. *Psychol. Aesthet. Creativity Arts*. **12**, 177 (2018).

[8] Darda, K. M. & Cross, E. S. The computer, A choreographer? Aesthetic responses to randomly-generated dance choreography by a computer. *Heliyon* **9** (2023). 10.1016/j.heliyon.2022.e12750 PMC9852657 36685468

[9] Eastwood, J., Snook, B. & Luther, K. What people want from their professionals: attitudes toward decision-making strategies. *J. Behav. Decis. Mak.* **25**, 458–468 (2012).

[10] Longoni, C., Bonezzi, A. & Morewedge, C. K. Resistance to medical artificial intelligence. *J. Consum. Res.* **46**, 629–650 (2019).

[11] Promberger, M. & Baron, J. Do patients trust computers? *J. Behav. Decis. Mak.* **19**, 455–468 (2006).

[12] Shaffer, V. A., Probst, C. A., Merkle, E. C., Arkes, H. R. & Medow, M. A. Why do patients derogate physicians who use a computer-based diagnostic support system? *Med. Decis. Mak*. **33**, 108–118 (2013). 10.1177/0272989X12453501 22820049

[13] Diab, D. L., Pui, S. Y., Yankelevich, M. & Highhouse, S. Lay perceptions of selection decision aids in US and non-US samples. *Int. J. Sel. Assess.* **19**, 209–216 (2011).

[14] Turel, O. & Kalhan, S. Prejudiced against the machine? Implicit associations and the transience of algorithm aversion. *MIS Q.* **47** (2023).

[15] Nadeem, A., Marjanovic, O. & Abedin, B. Gender bias in AI-based decision-making systems: a systematic literature review. *Aust. J. Inform. Syst.* **26** (2022).

[16] Drage, E., Mackereth, K. & Does AI debias recruitment? Race, gender, and AI’s eradication of difference. *Philos. Technol.* **35**, 89 (2022). 36246553 10.1007/s13347-022-00543-1 PMC9550152

[17] Tajfel, H. Experiments in intergroup discrimination. *Sci. Am.* **223**, 96–103 (1970). 5482577

[18] Billig, M. & Tajfel, H. Social categorization and similarity in intergroup behaviour. *Eur. J. Social Psychol.* **3**, 27–52 (1973).

[19] Deschrijver, E., & Ramsey, R. Unequal resource division occurs in the absence of group division and identity. *Proceedings of the National Academy of Sciences*. **122** (2025) 10.1073/pnas.2413797122 PMC11848414 39937852

[20] Bourhis, R. Y., Sachdev, I. & Gagnon, A. Intergroup research with the Tajfel matrices: Methodological notes. (1994).

[21] Logg, J. M., Minson, J. A. & Moore, D. A. Algorithm appreciation: people prefer algorithmic to human judgment. *Organ. Behav. Hum. Decis. Process.* **151**, 90–103 (2019).

[22] Burton, J. W., Stein, M. K. & Jensen, T. B. A systematic review of algorithm aversion in augmented decision making. *J. Behav. Decis. Mak.* **33**, 220–239 (2020).

[23] Heyselaar, E. The CASA theory no longer applies to desktop computers. *Sci. Rep.* **13**, 19693 (2023). 37952037 10.1038/s41598-023-46527-9 PMC10640629

[24] Bauer, K., von Zahn, M. & Hinz, O. Expl (AI) Ned: the impact of explainable artificial intelligence on users’ information processing. *Inform. Syst. Res.* **34**, 1582–1602 (2023).

[25] Allport, G. W. *The Nature of Prejudice*, vol. 2 (1954).

[26] Hsieh, T. Y., Chaudhury, B. & Cross, E. S. Human–robot cooperation in economic games: people show strong reciprocity but conditional prosociality toward robots. *Int. J. Social Robot.* **15**, 791–805 (2023).

[27] Ng, Y. L. When communicative AIs are cooperative actors: a prisoner’s dilemma experiment on human–communicative artificial intelligence Cooperation. *Behav. Inform. Technol.* **42**, 2141–2151 (2023).

[28] Nouri, E. & Traum, D. In *Human-Computer Interaction. Applications and Services: 15th International Conference, HCI International 2013, Las Vegas, NV, USA, July 21–26, 2013, Proceedings, Part II 15* 266–275 (Springer, 2013).

[29] Wu, J., Paeng, E., Linder, K., Valdesolo, P. & Boerkoel, J. C. In *2016 AAAI Fall Symposium Series.*

[30] Ishowo-Oloko, F. et al. Behavioural evidence for a transparency–efficiency tradeoff in human–machine cooperation. *Nat. Mach. Intell.* **1**, 517–521 (2019).

[31] McElreath, R. *Statistical Rethinking: A Bayesian Course with Examples in R and Stan* (Chapman and Hall/CRC, 2018).

[32] Barr, D. J., Levy, R., Scheepers, C. & Tily, H. J. Random effects structure for confirmatory hypothesis testing: keep it maximal. *J. Mem. Lang.* **68**, 255–278 (2013). 10.1016/j.jml.2012.11.001 PMC3881361 24403724

[33] Bürkner, P. C. brms: An R package for bayesian multilevel models using Stan. *J. Stat. Softw.* **80**, 1–28 (2017).

