# Supplementary material for: Comparing discriminatory behavior against AI and humans
Source: Sci Rep. 2025 Mar 29;15:10894. doi: 10.1038/s41598-025-94631-9 (PMC11954899; doi:10.1038/s41598-025-94631-9)
Supplement: Supplementary file 1 — Supplementary Material 1. [file 41598_2025_94631_MOESM1_ESM.docx]

**Supplementary Information**

| **Table S1. Survey Items** | | |
| --- | --- | --- |
| Construct | Items | Anchors |
| Attitude toward AI in general [1] | Based on your experience and/or familiarity with artificial intelligence (AI), please indicate the extent to which you agree with the following statements: (a) Using AI is a good idea, (b) Using AI is a wise idea, (c) Using AI is a desirable thing, (d) Using AI is beneficial | 1. Strongly Disagree  4. Neutral (neither agree nor disagree)  7. Strongly Agree |
| Trust in AI in general [2] | Please state your level of agreement with the following statements: (a) Even if not fully understood, I'd trust artificial intelligence tools to do a good job, (b) I trust artificial intelligence tools, (c) Artificial intelligence tools are trustworthy | 1. Strongly Disagree  4. Neutral (neither agree nor disagree)  7. Strongly Agree |
| Familiarity with AI [2] | To what extent… (a) ...are you familiar with the general AI concept? (b) ...are you familiar with specific AI applications? (c) ...are you using AI tools in your daily life? | 1. Strongly Disagree  4. Neutral (neither agree nor disagree)  7. Strongly Agree |
| Attitude toward the tested AI [1] | Based on your experience with the specific AI you rated, please indicate the extent to which you agree with the following statements about the specific AI: (a) Using this AI is a good idea, (b) Using this AI is a wise idea, (c) Using this AI is a desirable thing, (d) Using this AI is beneficial | 1. Strongly Disagree  4. Neutral (neither agree nor disagree)  7. Strongly Agree |
| Impulsivity [3] | Reflecting on the way you see yourself (not how you wish you were), please rate how much you agree with each of the following statements: (a) I say things without thinking, (b) I spend more money than I mean to, (c) I am often impatient, (d) I make “spur of the moment” decisions | 1. Strongly Disagree  4. Neutral (neither agree nor disagree)  7. Strongly Agree |
| Propensity to trust [4] | Reflecting on the way you see yourself (not how you wish you were), please rate how much you agree with each one of the following statements.  (a) One should be very cautious when working with other people. (b) Most people tell the truth about the limits of their knowledge. (c) Most people can be counted on to do what they say they will do. (d) If possible, it is best to avoid working with other people on projects. (e) Most people are honest in describing their experience and abilities. (f) Most people answer personal questions honestly.  (g) Most people are very competent in terms of their jobs. | 1 To no extent  5 To a great extent |

| **Table S2. Demographic Characteristics** | | | |
| --- | --- | --- | --- |
|  | | | |
|  |  | Human group  (n = 249) | AI group  (n=251) |
| Gender | Male | 127 | 121 |
|  | Female | 121 | 130 |
|  | Other | 1 |  |
|  | Prefer not to say |  |  |
| Education | Elementary school | 2 | 0 |
|  | High school | 32 | 23 |
|  | Some college or further | 36 | 43 |
|  | Trade or vocational school | 15 | 11 |
|  | Post-secondary certificate or diploma | 7 | 11 |
|  | Bachelor’s degree | 110 | 113 |
|  | Master’s degree | 36 | 36 |
|  | Doctorate | 11 | 14 |
| Age | Mean (years) | 39.8 | 40.1 |
|  | Stdev (years) | 13.9 | 14.7 |
| Handedness | Right | 214 | 211 |
|  | Left | 30 | 32 |
|  | Ambidextrous | 5 | 8 |

| **Table S3. Model bi (index) fixed effects in original units.** | | | | |
| --- | --- | --- | --- | --- |
| matrix_name | group | value | lower | upper |
| FAVvsMJP | human | 3.12 | 2.61 | 3.6 |
| FAVvsMJP | ai | 3.06 | 2.56 | 3.6 |
| MDvsMIP/MJP | human | 0.88 | 0.53 | 1.2 |
| MDvsMIP/MJP | ai | 1.13 | 0.72 | 1.5 |
| FAVvsP | human | 2.91 | 2.39 | 3.4 |
| FAVvsP | ai | 2.64 | 2.1 | 3.2 |

| **Table S4. Model bd fixed effects (factorial) in original units.** | | | |
| --- | --- | --- | --- |
| term | value | lower | upper |
| intercept | 2.484 | 2.16 | 2.79 |
| group | 0.047 | -0.52 | 0.606 |
| MDvsMIP/MJP | -2.206 | -2.65 | -1.771 |
| FAVvsP | -0.274 | -0.58 | 0.042 |
| group*MDvsMIP/MJP | 0.267 | -0.52 | 1.076 |
| group*FAVvsP | -0.219 | -0.83 | 0.391 |

**References**

1. Barki, H. & Hartwick, J. Measuring user participation, user involvement, and user attitude. *MIS quarterly*, 59-82 (1994).

2. Gefen, D. E-commerce: the role of familiarity and trust. *Omega* **28**, 725-737 (2000).

3. Tomko, R. L. *et al.* Measuring impulsivity in daily life: the momentary impulsivity scale. *Psychological assessment* **26**, 339 (2014).

4. Jarvenpaa, S. L., Knoll, K. & Leidner, D. E. Is anybody out there? Antecedents of trust in global virtual teams. *Journal of management information systems* **14**, 29-64 (1998).
